# Analyses of mitochondrial metabolism in diseases: a review on ^13^C magnetic resonance tracers

**DOI:** 10.1039/d4ra03605k

**Published:** 2024-11-27

**Authors:** Gaurav Sharma, Sergio Duarte, Qingyang Shen, Chalermchai Khemtong

**Affiliations:** a Department of Cardiovascular and Thoracic Surgery, University of Texas Southwestern Medical Center Dallas Texas USA; b Advanced Imaging Research Center, University of Texas Southwestern Medical Center Dallas Texas USA; c Department of Biomedical Engineering, The University of Texas Southwestern Medical Center Dallas Texas USA; d Department of Surgery, University of Florida Gainesville FL USA; e Department of Medicine, Division of Endocrinology, Diabetes and Metabolism, University of Florida Gainesville Florida USA chalermchai.khemtong@medicine.ufl.edu +1 (352) 273-8646; f Department of Biochemistry and Molecular Biology, University of Florida Gainesville Florida USA

## Abstract

Metabolic diseases such as obesity, type 2 diabetes, and cardiovascular diseases have become a global health concern due to their widespread prevalence and profound impact on life expectancy, healthcare expenditures, and the overall economy. Devising effective treatment strategies and management plans for these diseases requires an in-depth understanding of the pathophysiology of the metabolic abnormalities associated with each disease. Mitochondrial dysfunction is intricately linked to a wide range of metabolic abnormalities and is considered an important biomarker for diseases. However, assessing mitochondrial functions in viable tissues remains a challenging task, with measurements of oxygen consumption rate (OCR) and ATP production being the most widely accepted approaches for evaluating the health of mitochondria in tissues. Measurements of cellular metabolism using carbon-13 (or ^13^C) tracers have emerged as a viable method for characterizing mitochondrial metabolism in a variety of organelles ranging from cultured cells to humans. Information on metabolic activities and mitochondrial functions can be obtained from magnetic resonance (MR) analyses of ^13^C-labeled metabolites in tissues and organs of interest. Combining novel ^13^C tracer technologies with advanced analytical and imaging tools in nuclear magnetic resonance spectroscopy (NMR) and magnetic resonance imaging (MRI) offers the potential to detect metabolic abnormalities associated with mitochondrial dysfunction. These capabilities would enable accurate diagnosis of various metabolic diseases and facilitate the assessment of responses to therapeutic interventions, hence improving patient health and optimizing clinical outcomes.

## Abstract 2

This review discusses ^13^C NMR approaches for analyzing mitochondrial metabolism in tissues.

## Graphical abstract

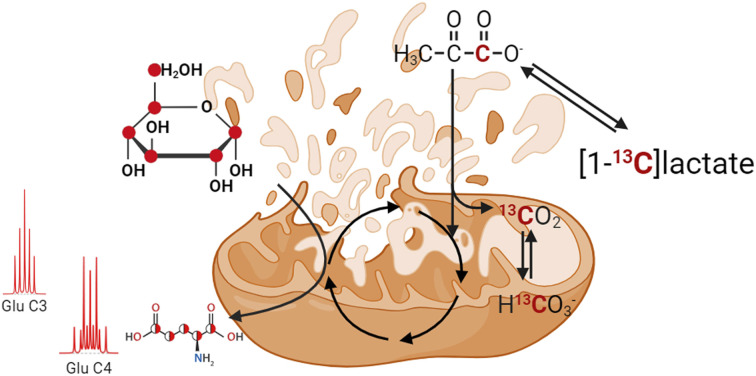

## 1. Introduction: metabolic diseases and mitochondrial dysfunction

Metabolic diseases such as obesity, type 2 diabetes, cardiovascular diseases, and metabolic dysfunction-associated steatotic liver disease (MASLD), formerly known as non-alcoholic fatty liver disease (NAFLD), have become a significant health concern^1,2^ with increasing prevalence worldwide.^3^ According to the World Health Organization (WHO), obesity incidence rates have nearly tripled since 1975, with approximately 2 billion and 650 million adults classified as overweight and obese, respectively.^4^ The prevalence of type 2 diabetes is also increasing globally, affecting hundreds of millions of adults worldwide.^5^ These metabolic diseases have a significant impact on life expectancy, quality of life, and the world's economy.^6^ It is therefore essential to develop effective strategies for the prevention and treatment of metabolic diseases to lessen the healthcare burden and, most importantly, improve the health of the global population.

**Fig. 1:**
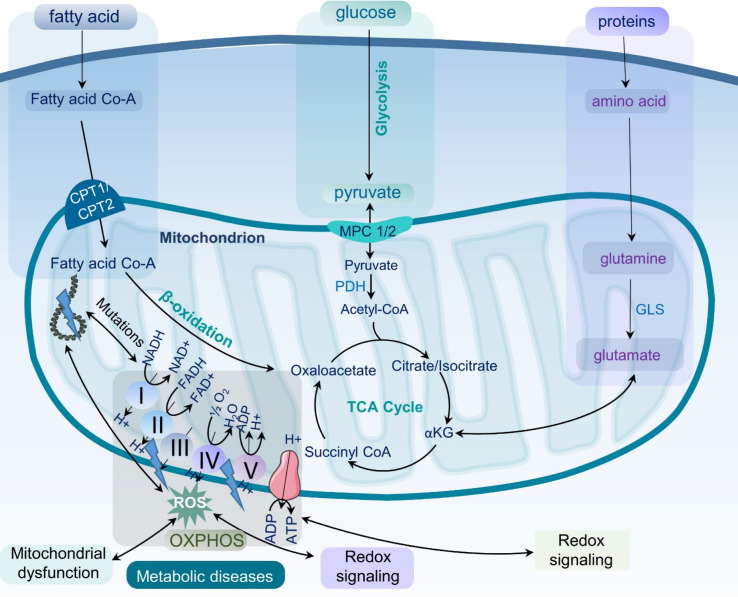
Mitochondrial metabolism and oxidative stress. Schematic representation of the role of mitochondrial metabolism in cellular function and the generation of reactive oxygen species (ROS). The figure also illustrates the effects of oxidative stress on mitochondrial function, including impaired energy production, altered signaling pathways, and the development of metabolic diseases. ROS – reactive oxygen species; ATP – adenosine triphosphate; OXPHOS – oxidative phosphorylation; TCA – the tricarboxylic acid cycle; PDH – pyruvate dehydrogenase; GLS – glutaminase.

**Fig. 2:**
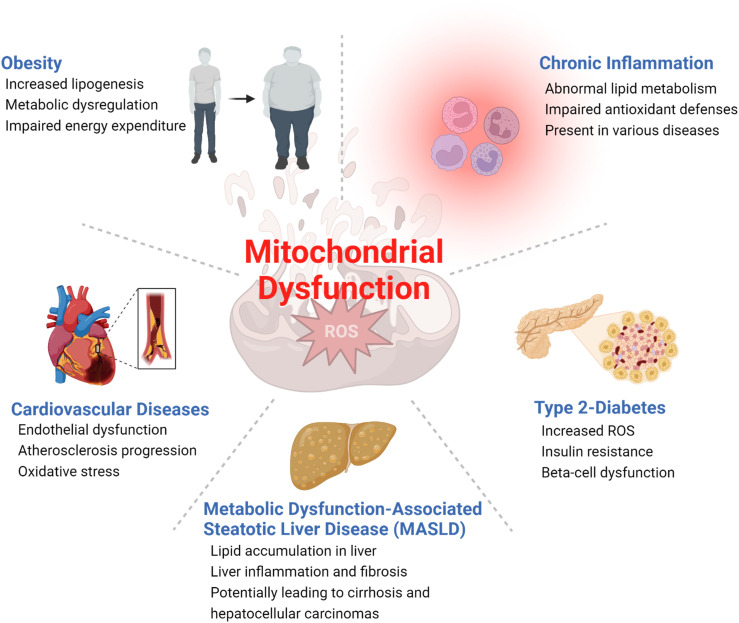
Examples of metabolic diseases associated with mitochondrial dysfunction.

Metabolic diseases are associated with dysfunctional mitochondria.^7^ Mitochondria are the powerhouse of cellular metabolism and play a critical role in energy production, apoptosis, calcium homeostasis, and signaling.^8^ They are essential for maintaining cellular homeostasis and ensuring cell survival and proliferation. The primary role of mitochondria is to generate adenosine triphosphate (ATP) through oxidative phosphorylation (OXPHOS), a process that involves electron transport chain (ETC) complexes I–IV and ATP synthase in the inner mitochondrial membrane (Fig. 1). Mitochondria also play key roles in other metabolic pathways, including the citric acid (TCA) cycle, β-oxidation of fatty acids, and amino acid metabolism.^9^ Last but not least, mitochondria play a critical role in the regulation and maintenance of cellular redox homeostasis.^10^ It is now well-accepted that mitochondrial dysfunction is an important hallmark of metabolic diseases including obesity, type 2 diabetes, cardiovascular diseases, MASLD, and inflammation (Fig. 2).^11,12^ Dysfunctional mitochondria are associated with alterations in mitochondrial metabolism and elevated levels of reactive oxygen species (ROS).^7^ The underlying mechanisms for mitochondrial dysfunction are complex but generally involve impaired mitochondrial biogenesis, alterations in mitochondrial membrane potential, altered mitochondrial dynamics, and increased production coupled with ineffective removal of ROS. Dysfunctional mitochondria have been linked to reduced glucose uptake, impaired insulin signaling, and altered lipid metabolism, which all contribute to the pathophysiology of metabolic diseases.^13^

Obesity and type 2 diabetes, two of the most common metabolic diseases affecting millions of people worldwide,^14^ have been associated with impaired mitochondrial functions with decreased oxidative metabolism and impaired glucose metabolism (Fig. 2). This metabolic shift, coupled with insulin resistance, leads to fat accumulations in the liver and skeletal muscles, among several other phenotypic changes.^15^ Moreover, dysregulations of mitochondrial ROS production have been associated with obesity and type 2 diabetes.^16,17^ Increased production of ROS impairs insulin signaling and causes oxidative damage to mitochondrial DNA and proteins, amplifying mitochondrial dysfunction and increasing risks of metabolic diseases. Mitochondrial dysfunction has also been associated with cardiovascular diseases.^18^ As a highly energy-demanding organ, the heart can be greatly affected by reduced ATP production and impaired cardiac function caused by mitochondrial dysfunction.^19,20^ Additionally, increased ROS in dysfunctional mitochondria can result in oxidative damages to cardiac cells, increasing the risk of heart failure. In the liver, mitochondrial dysfunction induces impaired β-oxidation of fatty acids, reduced ATP production, and increased ROS production, which all contribute to the development of MASLD,^21^ a common liver disease characterized by fat accumulation in the liver.^22^

Mitochondrial dysfunction has also been associated with inflammation. Increased and unregulated mitochondrial ROS production caused by the activation of immune cells such as macrophages can contribute to the development of both chronic and acute inflammatory diseases such as rheumatoid arthritis and pancreatitis.^23^ Sepsis is an acute and potentially life-threatening condition triggered by an extreme inflammatory response to infection that can lead to end-organ damage and failure.^24^ Sepsis is characterized by a complex interplay between mitochondrial dysfunction and immune and metabolic abnormalities. A hypermetabolic state is developed at the onset of sepsis, activating several catabolic processes such as lipolysis, proteolysis, ketolysis, and glycolysis. As sepsis progresses, these major metabolic alterations cause damages to mitochondrial integrity and functions, exacerbating mitochondrial DNA stress, oxidative damage to key enzymes, impaired oxidative phosphorylation, fission and fusion imbalances, impaired mitochondrial quality control machinery, and altered hormonal signaling. The combination of these complex cellular processes results in dwindling energy generation, abnormal inflammatory responses, and dysfunctional immune cells that subsequently lead to severe damages to vital organs.^25,26^ Eventual recoveries from sepsis are contingent on mitochondrial regeneration and restoration of adequate and normal physiologic bioenergetic pathways.^26^

Given the strong association of mitochondrial dysfunction with a wide spectrum of metabolic diseases, novel tools that accurately assess mitochondrial functions are needed for establishing in-depth understanding of disease pathogenesis as well as for developing effective targeted therapeutic interventions.^27^ Several methods are currently available for assessing mitochondrial functions, including high-resolution respirometry, enzymatic assays, and imaging techniques.^28,29^ However, certain limitations exist, such as the requirement for invasive procedures and *ex vivo* assays, compromising the practicality and accuracy of the assessments. The need for robust analytical and diagnostic tools for characterizing mitochondrial functions remains unmet and continuous efforts to develop novel tools for this purpose are therefore necessary.

## 2. Assessment of mitochondrial functions in viable tissues

**Fig. 3:**
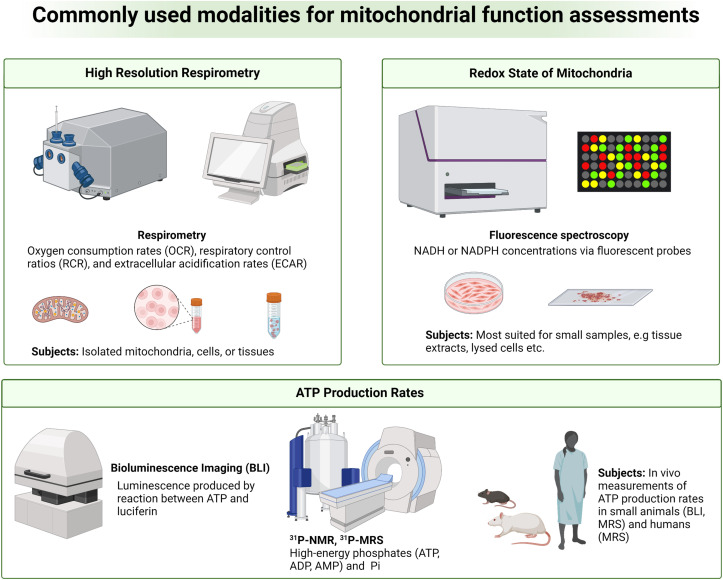
Overview of current methods for analyses of mitochondrial functions. Measurements of cellular respiration (top left), NADH or NADPH concentrations as a redox readout (top right), and ATP production rates (bottom) have been used to characterize mitochondria in various types of samples.

Despite the strong association between mitochondrial dysfunction and diseases, it is challenging to analyze abnormal mitochondrial functions as a biomarker for diseases due to the complex roles of mitochondria in cellular functions.^30^ While static determinations of free oxygen radicals, membrane potential, mitochondrial proteins and membrane integrity, and mitochondrial number and morphology can be used to indirectly assess mitochondrial quality, *in vivo* and real-time measurements of mitochondrial bioenergetics, dynamics, and metabolic fluxes provide a more direct and complete understanding of the state of mitochondrial functions.^31,32^ As mitochondria generate energy through OXPHOS, measuring oxygen consumption or respiration in tissues is one way to assess mitochondrial function. High-resolution respirometry (HRR) is a widely used and sensitive measurement of these parameters (Fig. 3, top left).^32^ Oxygen consumption rates (OCR), respiratory control ratios (RCR), and extracellular acidification rates (ECAR) are key metrics measured in HRR. Measurements of OCR, RCR, and ECAR can be done in real-time with an oxygen sensor in a closed chamber containing viable organelles such as isolated mitochondria, cells, and tissues. OCR is a measure of the rate of oxygen consumption by test samples, indicative of OXPHOS activity. RCR is an assessment of the degree of coupling between oxidative phosphorylation and ATP synthesis. ECAR is a rate of proton efflux from cells, which is indicative of glycolytic activity.^31,33^ Deviations from normal HRR measurements are indicative of mitochondrial dysfunction and are strongly associated with various diseases. Low OCR values denote inadequate mitochondrial ATP production, prospectively due to defects in the electron transport chain. Reduced RCR values convey that there is a lack of coupling between electron transport and ATP synthesis. Finally, elevations above normal ECAR values mark organelle's increased reliance on glycolysis, often a compensatory mechanism for inefficient mitochondrial ATP generation that further highlights mitochondrial dysfunction.

Different technologies and methods are available for HRR measurements. Some approaches rely on O_2_-dependent quenching of porphyrin-based phosphors (*e.g.* Seahorse XF Analyzer, Agilent Technologies Inc.) while others measure O_2_ consumption in viable samples with amperometric O_2_ sensors (*e.g.* Oroboros Oxygraph-2k, Oroboros Instruments). Seahorse XF Analyzers are a plate-based system with 3 fluorescent electrodes for measuring real-time O_2_ consumption and extracellular acidification.^34^ This approach was designed for high-throughput screenings with preserved samples' cellular integrity and generally requires a large number of cells or isolated mitochondria for reliable measurements.^35^ The Oroboros Oxygraph-2k is a widely used modular HRR instrument system that utilizes a polarographic O_2_ sensor to measure OCR over a range of O_2_ concentrations and temperatures. Oroboros Oxygraph-2k provides comprehensive, precise, and highly sensitive assessments of OXPHOS in isolated mitochondria, intact or permeabilized cells, and freshly prepared tissue slices.^36,37^ It is important to note that these approaches for assessing O_2_ metabolism in the context of mitochondrial function are primarily suited for *in vitro* and *ex vivo* analyses with limited practicality for *in vivo* measurements.

Another important hallmark of mitochondrial dysfunction is abnormal redox homeostasis, with unregulated production and release of ROS or imbalanced ratios of key molecular redox pairs such as NAD^+^/NADH, NADP^+^/NADPH, FAD/FADH_2_, and GSH/GSSG. Mitochondrial functions may therefore be assessed by analyzing the redox state of mitochondria.^38^ A variety of fluorescent probes have been developed over the years to measure NADH or NADPH concentrations. This strategy relies on chemical interactions between the fluorescent probes and the cofactors NADH or NADPH, producing a new fluorescent species with fluorescence emission distinguishable from the parent fluorescent probes.^38,39^ Examples of these probes include small molecular organic dyes (*e.g.* resorufin-based probes), genetically encoded protein-based probes (*e.g.* SoNar), and nanoparticle probes (*e.g.* carbon dots or metal complex-based probes).^40,41^ This technique is highly sensitive, both spatially and temporally, thanks to the excellent sensitivity of fluorescence spectroscopy. However, fluorescent probes face limitations in tissue penetration and therefore are most suited for smaller samples such as cultured cells and tissue slices (Fig. 3, top right). Autofluorescence originated from blood and tissues is another major drawback as significant signal interferences can be expected in highly perfused organs.^40^

ATP production rates are another index commonly measured for analyzing mitochondrial functions. In isolated mitochondria, cultured cells, and tissue slices, ATP production rates can be measured by bioluminescence spectroscopy in which firefly luciferase catalyzes the reaction between the newly produced ATP and luciferin to generate AMP, oxyluciferin, and quantifiable luminescent light (Fig. 3, bottom).^42^ Under the same principle, *in vivo* measurements of ATP production rates can be done in small animals such as mice and rats using bioluminescence imaging (BLI). Phosphorous-31 magnetic resonance spectroscopy (^31^P MRS) is another approach commonly used for measuring cellular ATP (Fig. 3, bottom).^10^ In this technique, ^31^P MR signal intensities of high-energy phosphates, *e.g.*, ATP and ADP, phosphocreatine (PCr), and inorganic phosphate (P_i_) can be quantified. Cellular bioenergetic states can then be analyzed from signal ratios of these metabolites such as ATP-to-ADP, ATP-to-P_i_, and ATP-to-PCr ratios. ^31^P MRS also allows for measurements of these high-energy metabolites' concentrations, which are useful parameters for mitochondrial function assessments. Additionally, ATP synthesis rates can be measured using ^31^P MRS. This is achieved by measuring the rates of PCr depletion coupled with ATP resynthesis, and *vice versa*, following ATP consumption by metabolic stress events, *e.g.*, exercise, immune activation, or drug administration.^43,44^ The PCr/ATP ratios measured by ^31^P MRS are also used as an indirect readout for mitochondrial oxidative capacity and cellular bioenergetic state.^45^^31^P MRS is a non-invasive technique, making it highly suitable for longitudinal measurements of cellular and mitochondrial functions where repeated data acquisitions are required. ^31^P MRS is a relatively insensitive method, like most magnetic resonance methods, and often requires lengthy data acquisition times. This drawback reduces temporal resolution and therefore hinders measurements of true ATP synthesis kinetics. MRS in general also suffers from poor spatial resolution. Analysis of a specific region of tissues or organs without signal contaminations from surrounding tissues is often impractical.^46,47^ Last but not least, changes in ATP production are not entirely mitochondria-specific and can be induced by other factors such as blood flow or oxygen delivery, complicating the use of ATP production as an index for assessing mitochondrial function.^47^

## 3. Assessment of intermediary metabolism with stable isotope carbon-13 tracers

As discussed earlier, altered intermediary metabolism is a metabolic phenotype associated with mitochondrial dysfunction. Measurements of energy metabolism could therefore reveal valuable information on the health of mitochondria and the disease state. Stable isotope carbon-13, used interchangeably with ^13^C or C-13, has emerged as a promising tracer for characterizing intermediary metabolism, potentially allowing for noninvasive assessments of mitochondrial functions in living tissues.^48–50^^13^C is non-ionizing and exists as a trace isotope (1.1% of all naturally occurring carbon isotopes), with ^12^C being the major isotope. Using metabolic substrates labeled with ^13^C, metabolism of the injected substrates can then be differentiated from that of endogenous sources by characterizing downstream metabolites using mass spectrometry (MS) or nuclear magnetic resonance (NMR) spectroscopy. MS is a powerful analytical method that measures molecular masses of chemical compounds, *i.e.*, metabolites in this case, enabling differentiations of ^13^C-labeled metabolites from those derived from non-^13^C-labeled endogenous substrates, thanks to the mass difference (∼1 atomic mass unit or amu) between ^13^C and ^12^C isotopes. MS is highly sensitive and can characterize small samples such as extracts of cultured cells and biopsied tissue samples. NMR is another method capable of analyzing ^13^C-labeling metabolites based on the intrinsic magnetic properties of the ^13^C isotope. It is important to note that ^12^C is not NMR-active and therefore cannot be directly detected by this technique. NMR is capable of not only detecting the presence of ^13^C-labeled metabolites but also of elucidating the ^13^C-labeling positions on the molecular structure of metabolites of interest. This unique capability makes ^13^C NMR highly suitable for analyzing intermediary metabolism in living tissues.

While both MS and NMR are widely used in metabolic analyses, this review will be primarily focused on NMR-based investigations of cellular metabolism using ^13^C tracers. This approach provides unique insights into metabolic fluxes, making it an indispensable tool for gaining a comprehensive understanding of mitochondrial functions.

### 3.1 Analyses of oxidative metabolism of isolated perfused organs by high-resolution ^13^C NMR

NMR is a powerful technique for chemical characterizations heavily utilized in synthetic chemistry. It has also played a key role in the characterization of small molecule metabolites as well as macromolecules such as proteins.^51^ In NMR, samples placed in a spectrometer operating at a high magnetic field are subjected to radiofrequency pulses that excite the nuclei of NMR-active atoms in the samples. The nuclei then relax and emit a radiofrequency signal that can be detected by the NMR spectrometer. The emitted radiofrequency is sensitive to the local electronic environment induced by chemical bonds around the nuclei, with resonances appearing over different ranges of chemical shifts on the NMR spectrum depending on the functional groups around the ^13^C nuclei. A downstream metabolite can be differentiated from its parent substrate based on unique chemical shifts of the two different chemical species. For example, ^13^C NMR resonances of glucose appear with chemical shifts ranging from around 60 ppm to 97 ppm on the ^13^C NMR spectrum while the resonances of its glycolytic product pyruvate appear at 27, 171, and 205 ppm. In principle, information on metabolic fluxes can then be obtained based on the disappearance and appearance rates of ^13^C MR intensities of glucose and pyruvate, respectively.^51^

**Fig. 4:**
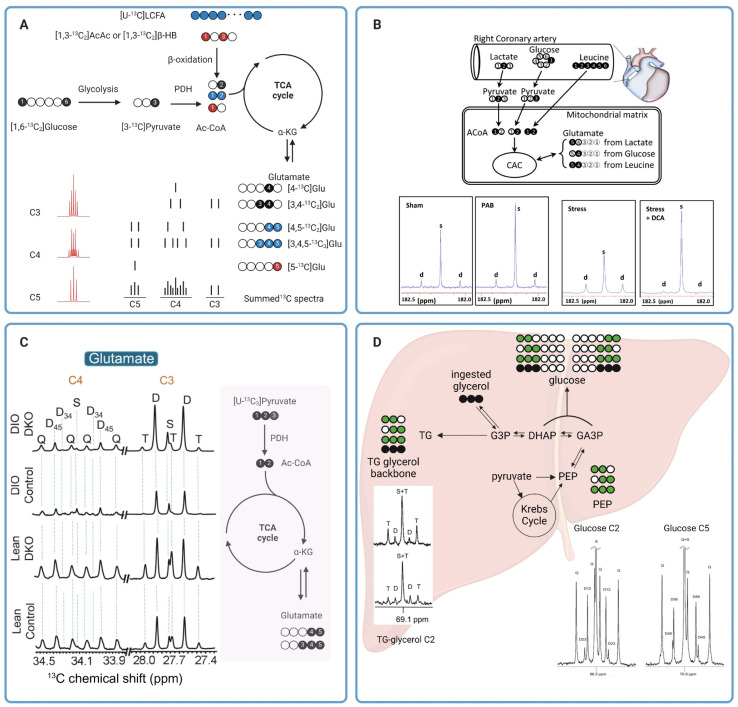
(A) A diagram showing production of various ^13^C glutamate isotopomers from different metabolic substrates and pathways. The ^13^C glutamate isotopomers along with a depiction of C3, C4, and C5 multiplets are shown in the lower right. For glutamate produced from glucose (black circles) and long chain fatty acids (blue circles), the isotopomer produced after the first and second turn of the TCA cycle are shown on the top and bottom drawing, respectively. The multiplets in the lower left were simulated using tcaSIM^55^ with 25%, 45%, and 20% of Ac-CoA originated from [U-^13^C]LCFA, [1,6-^13^C_2_]glucose, and [1,3-^13^C_2_]AcAc, respectively; (B) ^13^C tracer design for metabolic analysis of PAB-induced hypertrophied piglet hearts (top) and C5 glutamate resonance of tissue extracts (bottom);^59^ (C) C3 and C4 glutamate resonances of tissue extracts from lean or obese livers with and without PDK knock-out (left). A schematic showing production of ^13^C glutamate isotopomers from [U-^13^C_3_]pyruvate is shown in the right.^60^ (D) Analysis of hepatic metabolism in humans with [U-^13^C_3_]glycerol. Representative C2 and C5 resonances of glucose as well as C2 resonance of glycerol are also shown here.^62^

Despite its first use in organic chemistry in the late 1950s, ^13^C NMR was not utilized in metabolism applications until 1972 when it was used for analyzing the metabolism of ^13^C-enriched glucose in *saccharomyces cerevisiae*.^52^ About a decade later, Chance *et al.* proposed a method for estimating the TCA cycle flux in isolated perfused rat hearts using ^13^C fractional enrichments of glutamate as measured by ^13^C NMR of tissue extracts.^53^ Glutamate can be produced from the TCA cycle intermediate α-ketoglutarate (α-KG) *via* transamination, without altering the ^13^C labeling patterns between the two metabolites. It is more abundant than α-KG in tissues, making it an excellent metabolite for analyzing ^13^C labeling of the TCA cycle metabolites. Moreover, the five unique and distinguishable ^13^C resonances of glutamate appearing in a wide range on the NMR spectra (∼28–181 ppm) are also beneficial for the analysis of ^13^C multiplets arising from ^13^C–^13^C coupling. With five carbon centers in its skeletal molecular structure, glutamate has possible thirty-two isotope isomers, or isotopomers, as the number of isotopomers is equal to 2^5^.^54^ The different isotopomers include glutamate with ^13^C labeling patterns ranging from no ^13^C labeling at all, to those with only one ^13^C labeling at any of the five positions, and to the isotopomer with all five carbons labeled with ^13^C isotope (uniformly ^13^C-labeled glutamate or [U-^13^C_5_]glutamate). Different glutamate isotopomers can be produced depending on ^13^C labeling patterns of the oxidized metabolic substrates and how the substrates are metabolized into the TCA cycle. For example, β-oxidation of uniformly ^13^C-labeled long chain fatty acids ([U-^13^C]LCFA) (blue circles in Fig. 4A) produces uniformly ^13^C-labeled acetyl CoA ([U-^13^C_2_]Ac-CoA that fuses with oxaloacetate and, through several metabolites, subsequently produces the glutamate isotopomer with two ^13^C centers at C4 and C5 ([4,5-^13^C_2_]glutamate) following the first turn of the TCA cycle. ^13^C NMR spectra of this isotopomer has detectable C4 and C5 resonances, both as a doublet due to the ^13^C–^13^C coupling (C4D45 and C5D45). The second turn of the TCA cycle produces [3,4,5-^13^C_3_]glutamate. The C3 and C5 resonances of this isotopomer appear as a doublet due to the C3–C4 (C3D34) and C4–C5 (C5D45) spin–spin coupling, respectively. The C4 resonance appears as a doublet-of-doublets or quartet (C4Q) resulting from C4 coupling with both C3 and C5 (Fig. 4A). Oxidation of [1,3-^13^C_2_]acetoacetate ([1,3-^13^C_2_]AcAc) or [1,3-^13^C_2_]β-hydroxybutyrate ([1,3-^13^C_2_]βHB) into the TCA cycle produces [5-^13^C]glutamate (red circle) with a singlet peak appearing at the C5 resonance (C5S). Finally, metabolism of [1,6-^13^C_2_]glucose *via* glycolysis followed by oxidation into the TCA cycle produces [4-^13^C]glutamate after the first turn (C4S) and [3,4-^13^C_2_]glutamate (C4D34) after the second turn. The lower right panel of Fig. 4A shows drawings of individual multiplets for C3–C5 resonances as well as the summed spectra, demonstrating how multiplets from various spin–spin coupling for each resonance would appear in a^13^C NMR spectrum. The lower left panel of Fig. 4A shows ^13^C multiplets spectra simulated using tcaSIM^55^ with ^13^C-labeled Ac-CoA originated from various sources of substrates as follows: 25% [U-^13^C]LCFA, 45% [1,6-^13^C_2_]glucose, and 20% [1,3-^13^C_2_]AcAc) or [1,3-^13^C_2_]βHB. As discussed, simple ^13^C NMR analysis of glutamate ^13^C multiplets is a powerful technique for calculating the fractional oxidation of ^13^C-labeled metabolic substrates and estimating metabolic fluxes in tissues.^53,56^

In one notable example, Jones *et al.* applied NMR analysis of ^13^C glutamate isotopomers to evaluate effects of oxidative stress on energy metabolism in isolated perfused rat hearts.^57^ Oxidative stress was induced by exposing the perfused hearts to hydrogen peroxide or tertiary hydroperoxide. Results from the study showed that peroxide treatments led to a reduction in developed pressure. Real-time ^31^P NMR analysis of the perfused hearts showed a decrease in the ATP/PCr ratio in hearts exposed to hydrogen peroxide but not in hearts treated with tertiary butyl hydroperoxide. By comparing data from hearts receiving either [U-^13^C_6_]glucose combined with unlabeled acetate and lactate or [3-^13^C]lactate combined with [1,2-^13^C_2_]acetate and unlabeled glucose as oxidizable substrates, analysis of ^13^C enrichment of C3 and C4 of glutamate by high-resolution ^13^C NMR revealed that tertiary butyl hydroperoxide increased glucose oxidation into the TCA cycle while hydrogen peroxide treatment did not affect glucose oxidation. However, hydrogen peroxide significantly increased oxidation of exogenous lactate by the heart. Both oxidants caused a significant increase in carbohydrate oxidation *via* pyruvate dehydrogenase (PDH) (lactate and glucose combined) while suppressing oxidation of endogenous substrates, *i.e.*, triglycerides and glycogen, for energy production. In another example, Chatham *et al.* evaluated carbohydrate metabolism in Zucker diabetic fatty (ZDF) rat hearts.^58^ In this study, isolated hearts from ZDF rats were perfused with ^13^C-enriched carbohydrates; *i.e.* [U-^13^C_6_]glucose, [3-^13^C]lactate, and [2-^13^C]pyruvate, and unlabeled palmitate. Fractional oxidation of each ^13^C-labeled carbohydrate as well as nonlabelled substrates, palmitate and endogenous nutrients, can be estimated from ^13^C glutamate isotopomer analysis. Data from this study revealed a notable suppression of glucose oxidation into the TCA cycle through PDH in ZDF hearts, while oxidation of exogenous, ^13^C-labelled pyruvate and lactate between the healthy and diabetic hearts was comparable. Despite this shift in the metabolic phenotype, there were no detectable changes in systolic functions in the ZDF hearts compared with the healthy ones. This study highlights the premise that metabolic alterations often precede mechanical changes in cardiac functions and ^13^C tracers combined with high-resolution ^13^C NMR is a sensitive approach for evaluating these changes.

Similar approaches have also been applied to analyze mitochondrial metabolism in larger animals. Kajimoto *et al.* investigated metabolic reprogramming and altered carbohydrate oxidation in immature hypertrophied right ventricles in piglet hearts.^59^ Using a combination of [2-^13^C]lactate, [1-^13^C]glucose, and [U-^13^C_6_]leucine, the authors observed that immature hypertrophied right ventricle induced by pulmonary artery banding (PAB) favored oxidation of [2-^13^C]lactate as confirmed by the intense singlet peak compared to the doublet of glutamate C5 resonance (Fig. 4B, two left spectra) and ^13^C glutamate isotopomer analysis of right ventricle tissue extracts. In contrast, oxidation of [1-^13^C]glucose and [U-^13^C_6_]leucine into the TCA cycle was not different between the PAB and sham hearts. This study also revealed that infusion of dichloroacetate (DCA), a potent inhibitor of pyruvate dehydrogenase (PDK), amplified [2-^13^C]lactate oxidation through increased oxidation of pyruvate *via* PDH in immature hypertrophied right ventricle under hemodynamic stress induced by atrial pacing (Fig. 4B, two right spectra). The DCA-induced lactate oxidation was also accompanied by improved function of the hypertrophied right ventricle during stress. ^13^C NMR has also been applied to evaluate hepatic mitochondrial metabolism. In a recent study by our group, we have demonstrated that PDK knockout resulted in increased carbohydrate oxidation into the TCA cycle *via* PDH in both lean and diet-induced obese mice.^60^ Carbohydrate metabolism was evaluated in isolated perfused livers from lean and obese mice, with and without PDK knockout, with [U-^13^C_3_]pyruvate and [U-^13^C_3_]lactate used as carbohydrate sources. NMR analysis of ^13^C glutamate isotopomers revealed a lower degree of carbohydrate oxidation in obese mouse livers compared to those from lean animals as confirmed by lower fractional enrichment of [4,5-^13^C]glutamate (lower C4D45, Fig. 4C). PDK knockout increased carbohydrate oxidation in the liver of both lean and obese animals. The increased carbohydrate oxidation in obese, PDK knockout livers is also associated with a much lower degree of hepatic steatosis, emphasizing the accuracy of high-resolution ^13^C NMR for analyzing metabolic alterations due to diseases or treatments. In another example, Shannon *et al.* evaluated hepatic metabolism in obese mice with and without treatments with an insulin-sensitizing drug pioglitazone using high-resolution NMR.^61^ Following an intraperitoneal administration of [2,3-^13^C_2_]pyruvate to fasted animals, ^13^C isotopomer analysis of monoacetone glucose (MAG) chemically derivatized from plasma glucose revealed that pioglitazone treatments significantly reduced pyruvate-driven gluconeogenesis in obese mice. These mice had much higher fractional enrichments of [1,2-^13^C]glucose and [5,6-^13^C]glucose resulting from direct carboxylation of [2,3-^13^C_2_]pyruvate to oxaloacetate by PC followed by subsequent metabolism through gluconeogenic pathway. ^13^C-glutamate isotopomer analysis suggested that pioglitazone did not alter the balance between pyruvate metabolism through PC and PDH as evidenced by fractional enrichment of [2,3-^13^C]glutamate and [4,5-^13^C]glutamate, respectively, and despite the pioglitazone-induced decrease in gluconeogenesis from pyruvate. These findings demonstrate once again the benefits of high-resolution ^13^C NMR in the analyses of mitochondrial metabolism in viable tissues.

### 3.2 Probing *in vivo* metabolism in humans with high-resolution ^13^C NMR

Metabolic analyses such as those discussed in the previous section required tissue extracts for ^13^C NMR. The destructive nature of these methods renders their translation for metabolism measurements in humans impractical. Analyses of easily obtained samples like blood or plasma offer an alternative method for interrogating mitochondrial function. The non-invasive nature of these tests also allows for more frequent sampling, thereby enabling longitudinal assessments of the mitochondrial health. While this approach does not allow direct measurements of metabolic activities in tissues of interest, a careful selection of ^13^C-labeled substrates and subsequent downstream metabolites to be analyzed can provide us with vital insights into mitochondrial function of certain organs such as the liver and kidneys. As an example, Jin *et al.* demonstrated that NMR analyses of blood samples following oral administration of [U-^13^C_3_]glycerol is a viable approach for assessing the metabolic state and mitochondrial function in human livers *in vivo*.^62^ Fractional ^13^C enrichments of plasma glucose and the glycerol moiety of triglycerides hold vital information on *in vivo* metabolic fates of the ingested ^13^C-labeled glycerol, in the liver with different possible metabolic pathways, *e.g.* gluconeogenesis, the TCA cycle, and the pentose phosphate pathway (PPP). Direct utilization of [U-^13^C_3_]glycerol for glucose synthesis produces two glucose isotopomers, [1,2,3-^13^C_3_]glucose and [4,5,6-^13^C_3_]glucose. Oxidation of [U-^13^C_3_]glycerol into the TCA cycle *via* [U-^13^C_3_]pyruvate produces four potential isotopomers of glucose with ^13^C labeling of adjacent carbons at C1 and C2 ([1,2-^13^C_2_]glucose), C2 and C3 ([2,3-^13^C_2_]glucose), C4 and C5 ([4,5-^13^C_2_]glucose), or C5 and C6 ([5,6-^13^C_2_]glucose) positions. [1,2-^13^C_2_]Glucose can also be produced from metabolism of [U-^13^C_3_]glycerol to [1,2,3-^13^C_3_]glucose followed by metabolism *via* the PPP (Fig. 4D). Fractional ^13^C enrichments as obtained by analyzing multiplets of the C2 and C5 resonances of glucose can reveal the extent of [U-^13^C_3_]glycerol metabolism through the different metabolic pathways mentioned above. Meanwhile, direct esterification of exogenous [U-^13^C_3_]glycerol with free fatty acids results in triglycerides with ^13^C-labeling in all three positions of the triglyceride glycerol backbone. Double ^13^C-labeling of the glycerol moiety, *i.e.* [1,2-^13^C_2_]glycerol and [2,3-^13^C_2_]glycerol, is a result of [U-^13^C_3_]glycerol metabolism into the TCA cycle followed by gluconeogenesis. Metabolic fates of [U-^13^C_3_]glycerol can also be determined from fractional ^13^C enrichments of the glycerol backbone of triglycerides.

In this small pilot study, the authors employed [U-^13^C_3_]glycerol to analyze hepatic metabolism in healthy volunteers.^62^ Their results demonstrated that metabolism of exogenous [U-^13^C_3_]glycerol *via* the TCA cycle is stimulated by fasting. Doublet peaks at the C2 resonance (C2D) of the triglyceride glycerol moiety were detectable in ^13^C NMR spectra of plasma samples from both fed and fasted individuals, but higher in the fasted group (Fig. 4D). The presence of C2D of glycerol confirms the metabolism of [U-^13^C_3_]glycerol into the TCA cycle *via* [U-^13^C_3_]pyruvate followed by cataplerosis of oxaloacetate to produce [1,2-^13^C_2_]glycerol and [2,3-^13^C_2_]glycerol. Analyses of glucose ^13^C isotopomers confirmed the metabolism of exogenous [U-^13^C_3_]glycerol in the TCA cycle under both nutritional conditions, but higher under fasting, as corroborated by the presence of doubly ^13^C-labeled glucose, *i.e.* [1,2-^13^C_2_]glucose, [2,3-^13^C_2_]glucose, [4,5-^13^C_2_]glucose, [5,6-^13^C_2_]glucose (Fig. 4D). Higher metabolism of [U-^13^C_3_]glycerol *via* the PPP was also observed in fasted individuals as demonstrated by a bigger difference between [1,2-^13^C_2_]/[2,3-^13^C_2_] and [5,6-^13^C_2_]/[4,5-^13^C_2_] ratios in ^13^C NMR of plasma glucose. In a follow-up study, the authors investigated the impact of empagliflozin on [U-^13^C_3_]glycerol metabolism in hepatic gluconeogenesis of obese individuals without type 2 diabetes. ^13^C NMR analyses of plasma samples revealed that empagliflozin stimulated glucose synthesis from the exogenous [U-^13^C_3_]glycerol as confirmed by higher ^13^C incorporation in plasma glucose from subjects undergoing daily oral empagliflozin treatments. Empagliflozin did not affect glycerol metabolism *via* the PPP or TCA cycle. Metabolism of [U-^13^C_3_]glycerol for gluconeogenesis was also affected by visceral adipose tissue (VAT) levels, with lower ^13^C-labeling of plasma glucose observed in individuals with high VAT. These two studies have demonstrated that simple oral administrations of ^13^C-enriched glycerol may serve as a suitable tracer for interrogating hepatic metabolism in humans.

In another example, Fletcher *et al.* employed a different approach by combining a number of ^13^C-labeled metabolic tracers to analyze hepatic metabolism in 40 human subjects with varying hepatic triglyceride contents following a 24 hours fasting period.^63^ Regular metabolic analysis of the study subjects revealed that plasma ketones, acetoacetate (AcAc), and β-hydroxybutyrate (β-HB), were lower in subjects with MASLD than in the healthy control individuals. Among the MASLD patients, suppressed ketogenesis appeared to be exacerbated in those with higher hepatic triglyceride contents. Interconversion between the two ketones, as evaluated by co-infusing [3,4-^13^C_2_]AcAc and [1,2-^13^C_2_]β-HB and quantifying production of their respective metabolites, namely [3,4-^13^C_2_]β-HB and [1,2-^13^C_2_]AcAc, by high-resolution ^13^C NMR of plasma extracts, was impaired in MASLD patients regardless of hepatic triglyceride content levels. These findings likely reflect abnormal mitochondrial redox state or impaired activities of β-hydroxybutyrate dehydrogenase (BDH) enzyme in MASLD livers, both characteristics of mitochondrial dysfunction. Metabolic fates of acetyl-CoA were also evaluated by analyzing fractional ^13^C enrichment of plasma glucose following oral administration of [U-^13^C_3_]propionate.^64^ Their results revealed an altered acetyl-CoA disposal pathway and impaired ketogenesis, with metabolism into the TCA cycle as the primary fate for acetyl-CoA metabolism in MASLD patients despite prolonged fasting. Results from this study suggested that lipid disposal *via* oxidative metabolism into the TCA cycle and gluconeogenesis is favored over ketogenesis in MASLD livers, leading to elevated plasma glucose levels. Restoring ketogenesis may offer therapeutic potentials for MASLD, addressing disrupted lipid catabolism and glucose regulation.

As highlighted by the studies above, ^13^C NMR analyses of ^13^C metabolic tracers have a significant role in assessing mitochondrial functions in various biological systems. These studies have identified specific metabolic pathways that are altered in response to oxidative stress and mitochondrial dysfunction, providing valuable insights into the underlying mechanisms of metabolic alterations associated with diseases. With continued advancements in ^13^C metabolic tracer technologies, high-resolution NMR is expected to remain a vital tool for achieving further breakthroughs in the understanding of these important biological processes in the years ahead.

## 4. Real-time measurements of mitochondrial metabolism with hyperpolarized ^13^C MR

**Fig. 5:**
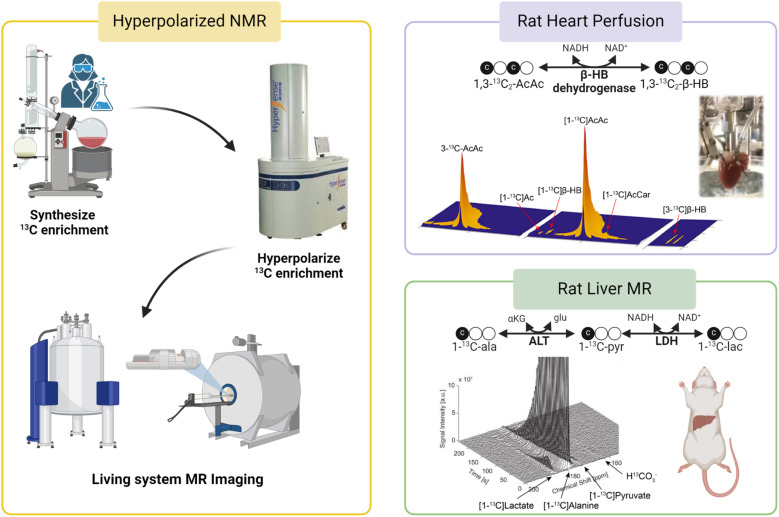
A workflow for HP ^13^C MR studies is shown in the left panel. (Top right) Metabolism of HP [1,3-^13^C_2_]AcAc to [1,3-^13^C_2_]β-HB as a mitochondrial redox probe;^71^ (bottom right) analysis of cellular redox in rat livers using HP [1-^13^C]Ala.^73^

In addition to its roles in metabolic flux analysis of tissue extracts, ^13^C MR has also been demonstrated as a novel method for evaluating cellular metabolism in real-time using magnetic resonance spectroscopy (MRS) or magnetic resonance imaging (MRI) techniques. For example, Park and coworkers showed that glycogen synthesis, a process called glycogenesis, can be detected real-time in human livers using ^13^C MRI following oral administration of a solution containing [1-^13^C]glucose.^65^^13^C MR signals of the liver clearly confirm delivery of the injected ^13^C-glucose showing an initial increasing trend during the first 30 min post ingestion followed by a gradual decrease in signal intensities. ^13^C intensity maps also showed the presence of both ingested [1-^13^C]glucose and newly synthesized ^13^C-labeled glycogen in the liver region, confirming the usefulness of ^13^C MRI for monitoring and quantifying utilization and metabolic fates of ^13^C-labeled tracers noninvasively in humans. It is important to note that despite the intrinsically poor sensitivity of detection of ^13^C MR, real-time detection of glycogen synthesis is feasible as demonstrated by this study due to the generally high glycogen concentrations in mammalian tissues (∼300 mM in the liver^66^). However, most metabolites of interest are present at a much lower concentration, reducing the practicality of ^13^C MRI for *in vivo* analyses of real-time metabolism of ^13^C-labeled tracers. The inherently poor sensitivity of ^13^C MRI must therefore be overcome. Hyperpolarized (HP) ^13^C MRI is an emerging noninvasive imaging technology that offers greatly improved ^13^C MRI sensitivity, enabling real-time assessments of metabolic processes in living organisms. This technique involves “polarization” of the ^13^C magnetic spins by dynamic nuclear polarization (DNP). DNP transfers polarization from highly polarized unpaired electrons to ^13^C nuclei through low-energy microwave irradiation at a cryogenic temperature (∼1 K), increasing the sensitivity of ^13^C MR by several orders of magnitude.^67,68^ HP ^13^C metabolic substrates produced by DNP can then be administered to test subjects and their metabolism can be detected by ^13^C NMR or MRI (Fig. 5, left). With the significantly improved MR sensitivity afforded by DNP, HP ^13^C MRI offers excellent temporal resolution and is capable of quantifying the utilization of injected ^13^C substrates and subsequent production of downstream metabolites with 1–2 seconds intervals. This technology allows for direct visualizations and quantification of metabolic activities and has significant potential as a diagnostic tool for characterizing metabolic diseases and oxidative stress *in vivo*.^69^

Since its introduction, HP ^13^C MR has been employed in the analysis of mitochondrial functions in metabolic diseases using various ^13^C-labeled substrates. In one example, Lee *et al.* used HP ^13^C MRI to determine changes in mitochondrial metabolism in a mouse model of high-fat diet induced type 2 diabetes by measuring real-time hepatic gluconeogenesis in the animals.^70^ The authors discovered that increased metabolism of HP [1-^13^C]pyruvate through pyruvate carboxylase (PC), as confirmed by increased signals of four-carbon metabolites [1-^13^C]malate and [1-^13^C]aspartate, is an important metabolic phenotype associated with increased hepatic glucose production in type 2 diabetes. This discovery suggests that HP ^13^C MRI enables the detection of metabolic changes during the development and progression of diabetes as well as longitudinal monitoring of liver function in diabetes. In principle, the metabolism of HP [1-^13^C]pyruvate *via* PDH can be analyzed as a readout for mitochondrial oxidative metabolism by quantifying signal intensities of the product of this metabolic reaction, ^13^CO_2_. However, ^13^CO_2_ signals are generally weak due to the extremely short T_1_ of ^13^CO_2_, coupled with the acid–base equilibrium that favors the enzymatic conversion of ^13^CO_2_ to its base-pair H^13^CO_3_^−^ at physiological pH. HP H^13^CO_3_^−^ is therefore a more suitable metabolite for this purpose and is generally analyzed as a readout for mitochondrial oxidation of HP [1-^13^C]pyruvate oxidation. Results from this study showed comparable normalized H^13^CO_3_^−^ signals in the livers of control and high-fat diabetic mice, suggesting that PDH fluxes were not different between the two groups of animals.

HP ^13^C MR has also been evaluated in the direct measurements of mitochondrial redox as a marker for mitochondrial dysfunction. Chen *et al.* used HP ^13^C NMR to investigate abnormal redox associated with mitochondrial dysfunction in isolated perfused rat hearts.^71^ Conversion of AcAc to β-HB is catalyzed by mitochondrial enzyme β-hydroxybutyrate dehydrogenase (BDH) and involves redox cofactors NAD^+^ and NADH. The authors hypothesized that the myocardial metabolism of HP ^13^C-AcAc to ^13^C-β-HB is sensitive to the mitochondrial NAD^+^/NADH ratio, a widely accepted measure for cellular redox (Fig. 5, top right). A higher conversion of ^13^C-AcAc to ^13^C-β-HB was observed in hearts subjected to ischemia or treatment with rotenone, a mitochondrial complex I inhibitor, than in the control hearts. This high production of β-HB in diseased myocardium reflects excess accumulation of mitochondrial NADH, indicative of imbalanced mitochondrial redox and dysfunctional mitochondria, induced by ischemia and pharmacological disruption of the electron transport chain. This study highlights the potential roles of HP ^13^C MR in the characterization of cellular redox and mitochondrial dysfunction in functioning hearts. In a follow-up study, Sharma *et al.* investigated the possibility of analyzing redox states of both the cytosolic and mitochondrial compartments simultaneously in isolated rat hearts using HP ^13^C MR.^72^ In this study, ^13^C-AcAc was co-polarized with [1-^13^C]pyruvate and injected into the perfused hearts. As discussed earlier, the metabolism of ^13^C-AcAc to ^13^C-β-HB is sensitive to mitochondrial NAD^+^/NADH ratios. Meanwhile, the production of [1-^13^C]lactate from HP [1-^13^C]pyruvate is catalyzed by the cytosolic enzyme lactate dehydrogenase (LDH), which requires cytosolic NAD^+^ and NADH, linking this process to the redox state of the cytosolic compartment. Simultaneous productions of HP ^13^C-β-HB and HP [1-^13^C]lactate as detected by ^13^C MR should therefore provide a readout for the redox state of both mitochondria and cytosols. Results from this study show that production of ^13^C-β-HB reflected changes in mitochondrial redox, consistent with the previous report, while metabolism of [1-^13^C]pyruvate to [1-^13^C]lactate was not sensitive to the altered redox state caused by ischemic and pharmacological interventions applied to the hearts under the conditions explored in that study.

In addition to the work discussed above, other ^13^C substrates and approaches have also been investigated as metabolic probes for real-time assessments of mitochondrial function by ^13^C MR. HP [1-^13^C]alanine has also been used to detect compromised mitochondrial function in rat livers following ethanol administration.^73^ Ratios of [1-^13^C]lactate to [1-^13^C]pyruvate were quantified in the liver of rats as a readout for mitochondrial redox for the organ (Fig. 5, bottom right). The authors reported that ethanol administration led to increased [1-^13^C]lactate/[1-^13^C]pyruvate ratios consistent with accumulated NADH and abnormal cellular redox in the liver induced by ethanol. While [1-^13^C]lactate/[1-^13^C]pyruvate ratios are thought to reflect the state of cytosolic redox, it is known that imbalanced cytosolic redox is linked to mitochondrial dysfunction^74,75^ and the authors correlated the increased [1-^13^C]lactate/[1-^13^C]pyruvate ratios in ethanol-treated animals to abnormal mitochondrial redox caused by metabolism of excess alcohol in these animals. Last but not least, a unique HP ^13^C probe [1-^13^C]dehydroascorbate (^13^C-DHA) was proposed as an imaging probe for evaluating cellular redox *in vivo*.^76,77^ HP ^13^C-DHA can be reduced to ^13^C-ascorbic acid (AA, vitamin C) requiring reduced glutathione (GSH) and NADPH. Both studies showed that HP ^13^C-DHA was readily reduced to ^13^C-AA in cancer cells and tumors. No oxidation of HP ^13^C-AA to ^13^C-DHA was observed in tumors when HP ^13^C-AA was used as the tracer, confirming a reduced redox state in tumor microenvironments.^77^ These studies demonstrated the benefits of HP ^13^C MR in interrogating mitochondrial function in real-time with ^13^C substrates. In addition to the HP ^13^C probes discussed in this section, other ^13^C agents have also been used to analyze various aspects of cellular metabolism and mitochondrial function. Review articles by Chaumeil *et al.*^78^ and Singh *et al.,*^79^ for example, are recommended for more in-depth discussions of this novel technology and ^13^C substrates for interrogating metabolic abnormalities in various diseases.

## 5. Conclusions and perspectives on future directions

There is a great need for analytical and diagnostic tools for assessing mitochondrial health given the strong correlation between mitochondrial dysfunction and metabolic diseases. Such capabilities to accurately obtain information on mitochondrial functions would greatly benefit the diagnosis and treatment of various metabolic diseases. Traditional methods currently employed for the assessments of mitochondrial functions offer varying degrees of clinical translational outlook, with ^31^P MRS being the most feasible modality for characterizing mitochondrial functions in a clinical setting. As mitochondrial dysfunction is associated with abnormalities in several metabolic pathways, assessments of mitochondrial functions can therefore be achieved by characterizing metabolic states of the organs or tissues of interest.

**Fig. 6:**
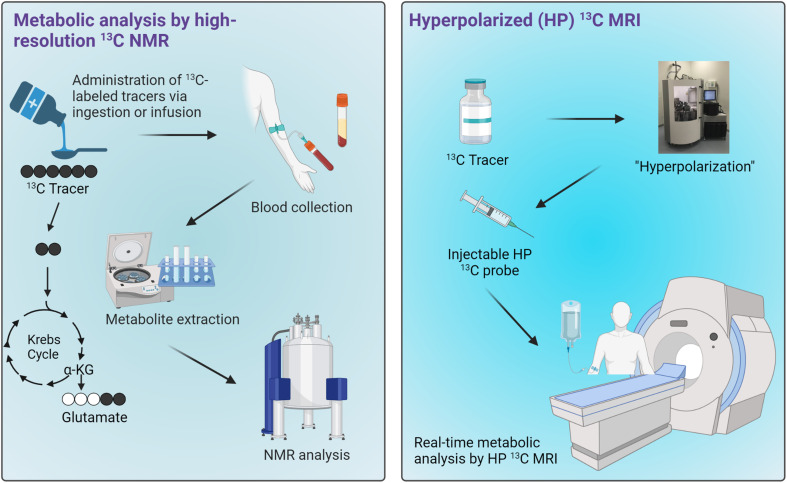
Potential applications of ^13^C tracers for metabolic analyses in humans. Workflows for metabolic analysis by high-resolution ^13^C NMR of blood samples (left) and HP ^13^C MRI (right).

^13^C-Tracers combined with NMR or MRI have been demonstrated as a novel technique for characterizing cellular metabolism through both invasive and noninvasive means, thus presenting a potential method for assessing mitochondrial functions in tissues. Following administration of ^13^C-labeled metabolic substrates such as carbohydrates and fats, downstream metabolites of the injected substrates can be labeled with ^13^C isotopes. It is important to note that while the metabolites of interest may be found in various cellular compartments as well as in the extracellular space, their production from the selected ^13^C-enriched tracers occurs specifically in the mitochondrial matrix. Therefore, the information on metabolic production of these ^13^C-metabolites is uniquely specific to the mitochondrial metabolic activities. Extracts of tissue or blood samples can be analyzed by high-resolution ^13^C NMR to obtain information on the metabolism of those substrates. This approach generally requires a relatively large sample size, several hundreds of milligrams, due to the inherently poor sensitivity of traditional ^13^C NMR. The technology has therefore been used mainly for measuring the metabolism in rodents, with only a select handful of studies carried out in humans focusing on analyses of plasma samples. This drawback is a major hurdle in the translation of this technology to the clinics. However, ^13^C NMR analysis of plasma is feasible and could present an ideal application for this technology, especially for metabolites that enter circulation in relatively high concentrations. One could envision a metabolic test that involves patients ingesting or being infused with a^13^C-labeled tracer cocktail followed by blood collection for ^13^C NMR analyses (Fig. 6, left). If an appropriate tracer that is sensitive to mitochondrial metabolism and produces downstream metabolites detectable in plasma can be established, a diagnostic assay using this approach could potentially be developed for assessing mitochondrial functions in the clinics.

Real-time metabolic analyses with HP ^13^C MRI have also been demonstrated in subjects ranging from rodents to humans over the past decade. HP ^13^C MRI offers a safe and rapid means to noninvasively assess mitochondrial function, potentially allowing for the detection of metabolic abnormalities associated with many diseases (Fig. 6, right). With the ability to detect metabolic changes, HP ^13^C MRI could be used to assess disease progression or monitor therapeutic responses given that these processes generally involve changes in cellular metabolism and mitochondrial functions. Despite the promise of HP ^13^C MRI, some limitations exist. One of the major hurdles to its clinical implementation is the complexity of data analysis it entails, necessitating specialized expertise in metabolic flux analysis and computational modeling. Moreover, special hardware is required for generating HP ^13^C substrates and for MRI acquisitions. Importantly, to date only [1-^13^C]pyruvate has demonstrated clinical applicability as a probe due to its favorably long HP ^13^C MR signal lifetime and rapid metabolism *in vivo*. Research on several other probes is underway, with results from human imaging studies expected over the next few years. The future clinical utility of this innovative technology will rely heavily on the diagnostic capabilities of HP [1-^13^C]pyruvate across various pathologies, as well as the development and emergence of new probes that are both clinically feasible and diagnostically valuable.

In conclusion, ^13^C metabolic tracing by MR is a powerful approach for measuring mitochondrial metabolism in living organelles. Much work remains to be done to establish important metabolic biomarkers that are assessable by ^13^C MR tracers for effective translation of these novel technologies to the clinics and establish them as valuable tools for the management and treatment of metabolic diseases.

## Data availability

No new data were analyzed or included in this review article.

## Author contributions

All authors were involved in the conceptualization and writing of this manuscript. Funding was acquired by GS, SD, and CK.

## Conflicts of interest

The authors have no conflicts to declare.
